# CIEC: Cross-tissue Immune Cell Type Enrichment and Expression Map Visualization for Cancer

**DOI:** 10.1093/gpbjnl/qzae067

**Published:** 2024-10-03

**Authors:** Jinhua He, Haitao Luo, Wei Wang, Dechao Bu, Zhengkai Zou, Haolin Wang, Hongzhen Tang, Zeping Han, Wenfeng Luo, Jian Shen, Fangmei Xie, Yi Zhao, Zhiming Xiang

**Affiliations:** Central Laboratory, The Affiliated Panyu Central Hospital of Guangzhou Medical University, Guangzhou 511400, China; Shenzhen Engineering Center for Translational Medicine of Precision Cancer Immunodiagnosis and Therapy, YuceBio Technology Co., Ltd., Shenzhen 518081, China; Shenzhen Engineering Center for Translational Medicine of Precision Cancer Immunodiagnosis and Therapy, YuceBio Technology Co., Ltd., Shenzhen 518081, China; Research Center for Ubiquitous Computing Systems, Institute of Computing Technology, Chinese Academy of Sciences, Beijing 100190, China; School of Management, Beijing University of Chinese Medicine, Beijing 100029, China; School of Management, Beijing University of Chinese Medicine, Beijing 100029, China; Shenzhen Engineering Center for Translational Medicine of Precision Cancer Immunodiagnosis and Therapy, YuceBio Technology Co., Ltd., Shenzhen 518081, China; Central Laboratory, The Affiliated Panyu Central Hospital of Guangzhou Medical University, Guangzhou 511400, China; Central Laboratory, The Affiliated Panyu Central Hospital of Guangzhou Medical University, Guangzhou 511400, China; Central Laboratory, The Affiliated Panyu Central Hospital of Guangzhou Medical University, Guangzhou 511400, China; Central Laboratory, The Affiliated Panyu Central Hospital of Guangzhou Medical University, Guangzhou 511400, China; Research Center for Ubiquitous Computing Systems, Institute of Computing Technology, Chinese Academy of Sciences, Beijing 100190, China; Central Laboratory, The Affiliated Panyu Central Hospital of Guangzhou Medical University, Guangzhou 511400, China

**Keywords:** Cancer single-cell data, Cross-tissue analysis, Immune cell, Enrichment analysis, Expression map

## Abstract

Single-cell transcriptome sequencing technology has been applied to decode the cell types and functional states of immune cells, revealing their tissue-specific gene expression patterns and functions in cancer immunity. Comprehensive assessments of immune cells within and across tissues will provide us with a deeper understanding of the tumor immune system in general. Here, we present Cross-tissue Immune cell type or state Enrichment analysis of gene lists for Cancer (CIEC), the first web-based application that integrates database and enrichment analysis to estimate the cross-tissue immune cell types or states. CIEC version 1.0 consists of 480 samples covering primary tumor, adjacent normal tissue, lymph node, metastasis tissue, and peripheral blood from 323 cancer patients. By applying integrative analysis, we constructed an immune cell type/state map for each context, and adopted our previously developed Kyoto Encyclopedia of Genes and Genomes (KEGG) Orthology Based Annotation System (KOBAS) algorithm to estimate the enrichment for context-specific immune cell types/states. In addition, CIEC also provides an easy-to-use online interface for users to comprehensively analyze the immune cell characteristics mapped across multiple tissues, including expression map, correlation, similar gene detection, signature score, and expression comparison. We believe that CIEC will be a valuable resource for exploring the intrinsic characteristics of immune cells in cancer patients and for potentially guiding novel cancer–immune biomarker development and immunotherapy strategies. CIEC is freely accessible at http://ciec.gene.ac/.

## Graphical Abstract

**Graphical Abstract:**
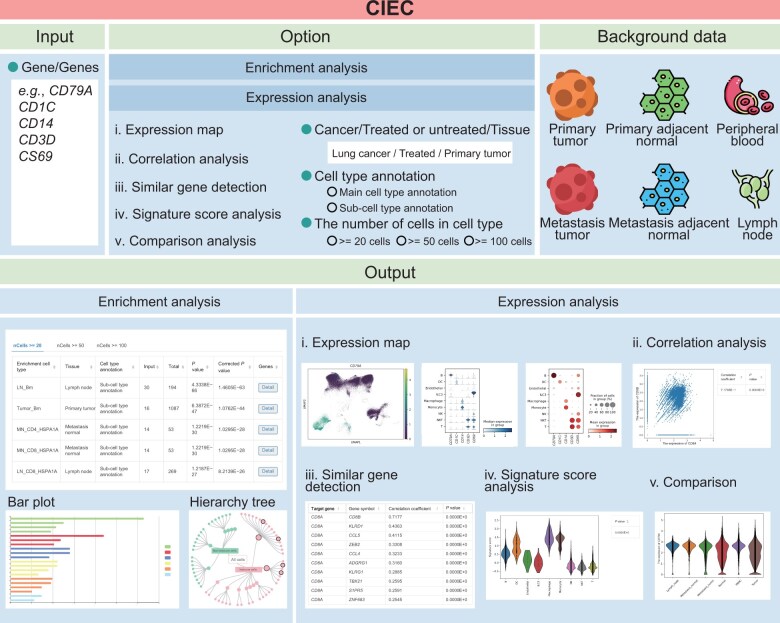

## Introduction

The tremendous advances in single-cell RNA sequencing (scRNA-seq) technologies have enabled deep insight into the highly complex tumor heterogeneity and diversity in immune cell types and states [1,2]. With the massive accumulation of cancer-related scRNA-seq data, several databases and web servers have been developed. For instance, Cancer Single-cell State Atlas (CancerSEA) [3] provides the functional states of cancer cells at the single-cell level across 25 types of cancer. Cancer Single-cell Expression Map (CancerSCEM) [4] has collected and annotated scRNA-seq datasets encompassing 20 types of cancer and provided analytical capabilities in gene and sample, as well as other search functions. Despite these efforts, large-scale cross-tissue studies that investigate tissue-specific features of immune cells in cancer patients have not been reported. Recently, a growing appreciation of immune diversity across tissues in cancer research has emerged [5,6]. For example, exhausted T cells as a group of dysfunctional T cells were preferentially enriched in tumor tissues, while effector T cells were prevalent in the peripheral blood of cancer patients [6]. Given that immune cells are composed of many different cell types distributed in various tissues that act together to mediate protective function, understanding anti-tumor immunity requires a comprehensive assessment of the features and properties of immune cells within and across tissues.

Moreover, a fundamental challenge to performing the cross-tissue analysis is integrative immune cell type annotation, including identifying rare immune cell subgroups and distinguishing novel types from previously well-annotated cell groups. In this context, a comprehensive catalog of immune cell types identified across tissues is required for an in-depth dissection of the heterogeneity of immune cells.

**Figure 1:**
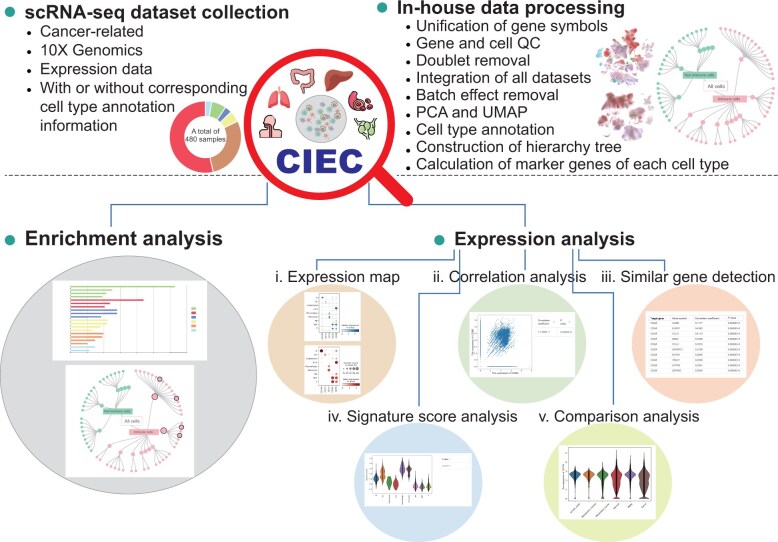
Overview of CIEC Based on cross-tissue scRNA-seq data integration and processing, CIEC provides two major functional modules for characterizing cross-tissue immune cells, including enrichment and expression analyses. To facilitate exploration and visualization, a user-friendly web interface for CIEC was developed, enabling users to analyze, browse, search, and download analytical results of interest. CIEC, Cross-tissue Immune cell type or state Enrichment analysis of gene lists for Cancer; scRNA-seq, single-cell RNA sequencing; QC, quality control; PCA, principal component analysis; UMAP, uniform manifold approximation and projection; DC, dendritic cell; NK, natural killer; NKT, natural killer T; ILC3, type 3 innate lymphoid cell; PBMC, peripheral blood mononuclear cell.

To address these needs, we developed Cross-tissue Immune cell type or state Enrichment analysis of gene lists for Cancer (CIEC) (**Figure 1**), the first web-based application for cross-tissue immune cell type enrichment and expression map visualization for cancer. CIEC version 1.0 collected millions of high-quality single cells from 480 samples across five tissue types of cancer patients (including primary tumor, adjacent normal tissue, metastasis tumor, lymph node, and peripheral blood). Through the integration of scRNA-seq data and in-depth analyses, CIEC offers enrichment analysis for immune cell types across tissues and gene expression analyses that include five subfunctions. We believe that CIEC will help researchers gain new insights into how tumor–immune systems work and will be widely adopted in cancer research.

## Database content and features

### Framework of CIEC

**Figure 2:**
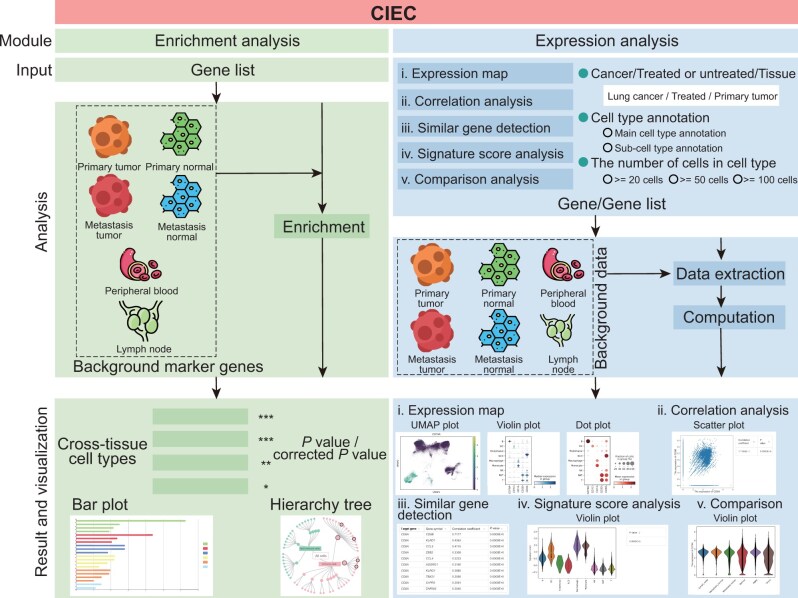
Schematic overview of the CIEC workflow CIEC provides two major modules including “enrichment analysis” and “expression analysis”. The enrichment analysis only requires a gene symbol list or a gene list file as the input. The expression analysis consists of five subfunctions, including “expression map”, “correlation analysis”, “similar gene detection”, “signature score analysis”, and “comparison analysis”. Various tables and figures are provided in CIEC for visualization of the results.

The current CIEC consists of two parts, namely, the “enrichment analysis” module and the “expression analysis” module (**Figure 2**). The enrichment analysis module gives an answer about which cell types and tissue types are statistically significantly associated with the input gene list, which only requires a gene symbol list or a gene list file as the input. A task link is provided for each task for the user to fetch results directly now and in the future. These inputted gene symbols will automatically be mapped to our background marker gene symbols. The expression analysis module provides five subfunctions, including “expression map”, “correlation analysis”, “similar gene detection”, “signature score analysis”, and “comparison analysis”.

### Enrichment analysis and example outputs

**Figure 3:**
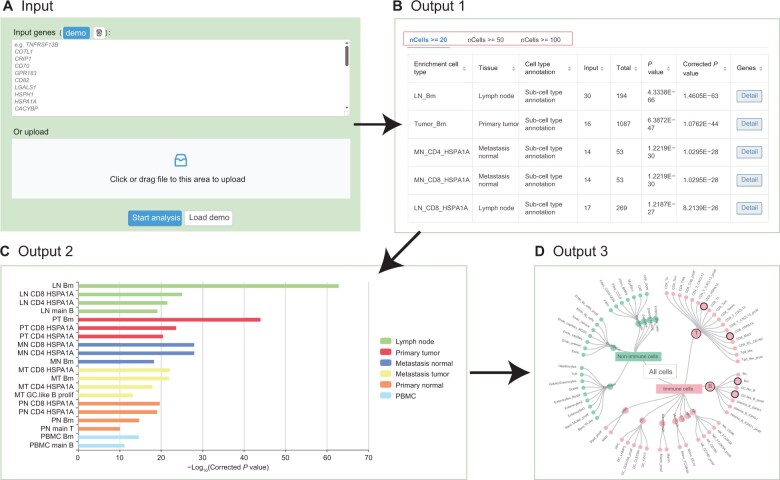
Enrichment analysis interface overview for illustrating an example result visualization **A**. Input page. **B**. Screenshot of a portion of the tabular display of enriched cell types. **C**. Bar plot showing the top 20 enriched cell types ranked by corrected *P* values (−log_10_ transformed). **D**. Hierarchy tree showing the projection of the top 20 enriched cell types onto the entire cell types. PT, primary tumor; PN, primary normal; MN, metastasis tumor; MT, metastasis normal; LN, lymph node; Tn, naïve T; Tcm, central memory T; Treg, regulatory T; Tem, effector memory T; Temra, recently activated effector memory T; MAIT, mucosal-associated invariant T; IEL, intraepithelial lymphocyte; Tgd, gamma delta T; Bn, naïve B; Bm, memory B; GC, germinal center; CAF, cancer-associated fibroblast; TA, transit-amplifying.

The enrichment analysis page enables users to utilize custom gene sets to perform cross-tissue immune cell type enrichment analysis. Based on the Kyoto Encyclopedia of Genes and Genomes (KEGG) Orthology Based Annotation System (KOBAS) enrichment algorithm [7,8], Chi-square or Fisher’s exact test is used to determine the association between the inputted gene set and the cross-tissue cell type-specific marker genes. After inputting a gene list and clicking the “Start analysis” button on the enrichment analysis page, the query results including three panels will be displayed (**Figure 3**A). The first panel displays the detailed cross-tissue enrichment results (Figure 3B). Different results will be obtained by applying different cell count filtration criteria (20, 50, and 100 cells) (Figure 3B). The second panel displays the top 20 enriched cell types according to the ranked corrected *P* values (−log_10_ transformed) by a bar chart (Figure 3C). The third panel displays the projection of the top 20 enriched cell types onto the hierarchy tree (Figure 3D). All these results provide users with a clear and structured summary of the cross-tissue enrichment analysis of their custom gene set.

### Expression analysis and example outputs

**Figure 4:**
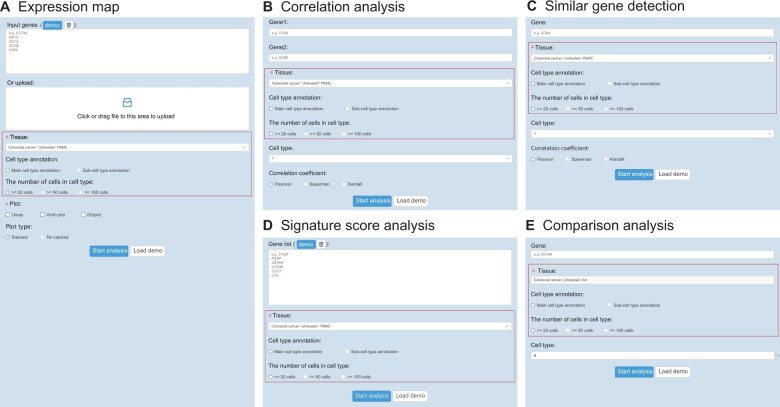
Input pages for five subfunctions in the expression analysis module **A**. Input page of the expression map. **B**. Input page of the correlation analysis. **C**. Input page of the similar gene detection. **D**. Input page of the signature score analysis. **E**. Input page of the expression comparison. The red boxes indicate similar checkbox options in each subfunction.

**Figure 5:**
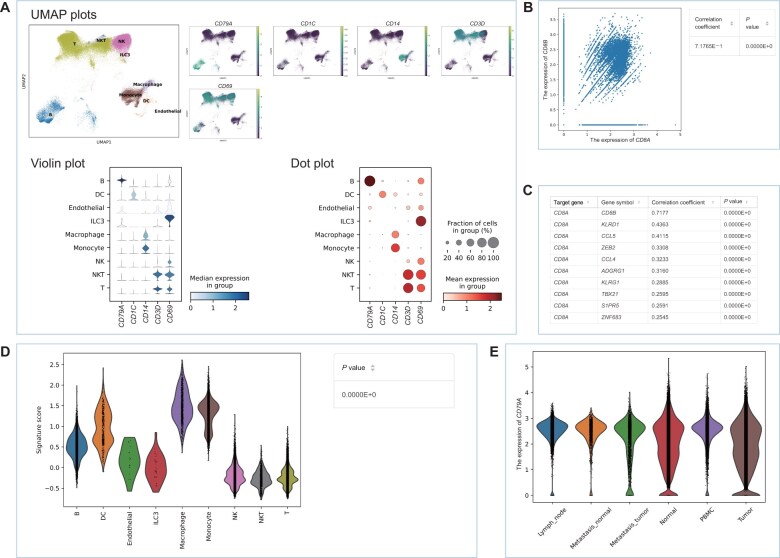
Output pages for five subfunctions in the expression analysis module **A**. Expression map: UMAP plot, dot plot, or violin plot showing the expression of input genes in specific contexts. **B**. Correlation analysis: scatter plot and table showing the correlation between two input genes in specific contexts. **C**. Similar gene detection: table showing the top 10 genes most correlated with the input gene in specific contexts. **D**. Signature score analysis: violin plot showing the signature scores of input gene list in specific contexts. **E**. Comparison analysis: violin plot showing the expression distribution of the input gene across tissues in specific contexts.

To provide users with systematic and comprehensive cross-tissue immune cell analyses, we developed five subfunctions in the expression analysis module, including “expression map”, “correlation analysis”, “similar gene detection”, “signature score analysis”, and “comparison analysis”. Similar checkbox options were provided for users in each subfunction (**Figure 4**A–E). On each page of subfunctions, after clicking the “Start analysis” button, query results will be displayed. With the expression map function, users can efficiently explore the expression profiles of genes of interest, providing an accurate representation of gene expression within specific contexts (Figure 4A). In addition, users can select one or multiple items to display by uniform manifold approximation and projection (UMAP) plots, violin plots, and/or dot plots (**Figure 5**A). Correlation analysis allows users to obtain correlation results between two genes of their interest under specific contexts (Figure 4B). Here, we provide users with an overview of the correlation analysis results of two query genes by a scatter plot, along with the corresponding correlation coefficient and *P* value (Figure 5B). Three methods of correlation coefficient calculation, such as Pearson correlation, Spearman correlation, and Kendall correlation, are provided for users to choose from (Figure 4B). The similar gene detection function displays all genes correlated with the target gene in specific contexts by the tabular (Figure 5C). To conduct a meaningful signature score analysis, we suggest that users input the gene symbol list with a length greater than 2. The signature score is obtained by calculating the average expression of input gene list subtracted by the average expression of all background genes (Figure 4D). Results are illustrated by violin plots along with the corresponding *P* value among multiple groups (Figure 5D). Finally, the comparison analysis function permits users to compare the expression profiles of genes of interest in specific contexts across cancer tissue types (Figure 4E), and the result is illustrated by a violin plot (Figure 5E).

## Discussion

In this work, we carried out a large-scale cross-tissue study to investigate tissue-specific features of immune cells in cancer patients and developed a database and web server for cross-tissue immune cell type enrichment and expression map visualization. CIEC provides an easy-to-use interface for users to comprehensively analyze the immune cell characteristics mapped across multiple tissues. Nevertheless, the following areas are targeted for future improvements on this web server: (1) the current version mainly focuses on gastrointestinal and respiratory cancer types, and expanding to cover more cancer types and corresponding tissue types is needed to further enrich our database and web server in the further update of the website; (2) integrating with other single-cell sequencing data, such as spatial transcriptomics and single-cell epigenomic data, to better investigate the cross-tissue immune cell profiles; (3) adding more treatment information, such as clinical treatment modalities and prognostic information, to extend the value of our database, and (4) establishing a flexible and extensible framework so that users can conveniently analyze and submit their data to the database. As the first cross-tissue single-cell analysis application, we believe that CIEC will be a valuable resource for exploring the intrinsic characteristics of immune cells in cancer patients and could support users to perform follow-up studies to further explore the mechanisms of anti-tumor immunity.

## Method

### Cancer scRNA-seq data collection and processing

To conduct a comprehensive and systematic integrated analysis of cancer at the single-cell resolution, millions of cancer scRNA-seq data were downloaded from the Gene Expression Omnibus (GEO, http://www.ncbi.nlm.nih.gov/geo), including raw or processed expression matrices and corresponding cell type annotation information [6,9–20] (Table S1). Briefly, a total of 1,730,501 cells covering 480 samples were collected, including primary tumor, lymph node, metastasis tissue, adjacent normal tissue (primary normal tissue derived from the normal tissue where the primary tumor is located, and metastasis normal tissue derived from the normal tissue where the metastasis tumor is located), and peripheral blood. Samples associated with clinical treatment were classified as treated and untreated groups. Users can select the relevant labels from drop-down menus.

### Cell quality control, unsupervised clustering, and cell type annotation

The Python (v3.8.8) package Scanpy (v1.9.1) was used for scRNA-seq data analysis. For each dataset, genes that were detected in fewer than 3 cells were filtered out. Cells with fewer than 600 or greater than 6000 detected genes and a high detection rate (20%) of mitochondrial gene expression were filtered out. To remove the potential doublets in these data, the “scanpy.external.pp.scrublet” function in Scanpy was used with the parameters: expected_doublet_rate = 0.03 and threshold = 0.25. Finally, a total of 1,215,554 high-quality single cells were retained. The distribution of samples and cells across tissues in different cancer types is shown in Figure S1.

After quality control, we applied the “scanpy.AnnData.concatenate” function with the parameter “join” set to “outer” to merge all scRNA-seq data. Then, the “scanpy.pp.highly_variable_genes” function was utilized to annotate highly variable genes. The “scanpy.tl.pca” function with the parameter “svd_solver” set to “arpack” was used to calculate the principal component analysis (PCA) coordinates, loadings, and variance decomposition. The first 50 components were used for further analysis. The batch effect across different individuals was removed by the Harmony algorithm with the parameter “batch_key” set to “Patient”. The “scanpy.tl.umap” function was used to reduce the dimensionality. The “scanpy.tl.louvain” function was adopted to cluster single cells based on an unsupervised graph-based clustering algorithm.

For cell type annotation, the clustering of all cells was performed at resolution = 2 and then annotated based on the specific markers of major immune cell types (including T cells, B cells, natural killer cells, and myeloid cells) and non-immune cell types (including epithelial cells, endothelial cells, and fibroblasts) (Table S2). The major cell types were extracted one by one for sub-cell type clustering and annotation. Finally, a total of 68 cell types including immune and non-immune cell subtypes were identified, and a comprehensive cell type map for each tissue type was constructed. The “scanpy.tl.rank_genes_groups” function was used to calculate the marker genes of all tissue-specific cell types.

### Cross-tissue cell enrichment analysis

We applied our previously developed KOBAS enrichment algorithm to estimate the enrichment of context-specific immune cell types. Specifically, we implemented a two-step procedure that involves counting foreground and background gene sets, followed by calculating the number of marker genes and non-marker genes for each enriched cell type. Subsequently, a statistical analysis involving either the Chi-square test or Fisher’s exact test was conducted to determine the significance of the enrichment and to provide a corresponding *P* value. The method presented in this study offers a rigorous approach to identifying highly enriched cell types in cross-tissue analyses, with potential applications in various biological domains.

### Database and web service design

The CIEC website is powered by the NGINX (v1.14.1) service and uses a MySQL (v5.7.40) database to store data. Methods are refactored into RESTful APIs, and data are dynamically updated on the web page using AJAX (v3.0.0). To improve accessibility, REACT (v16.8.6) frontend framework is adopted, while Ant Design (v3.19.5) component library is used to build user-friendly layouts and display data tables, and ECharts (v4.8.0) is utilized for interactive chart display.

## Supplementary Material

qzae067_Supplementary_Data

## Data Availability

CIEC is a free and publicly available web tool that can be accessed online at http://ciec.gene.ac/.

## CRediT author statement

**Jinhua He:** Formal analysis, Visualization, Writing – original draft. **Haitao Luo:** Conceptualization, Writing – original draft, Writing – review & editing, Supervision. **Wei Wang:** Formal analysis, Visualization, Writing – original draft. **Dechao Bu:** Visualization, Software, Writing – original draft. **Zhengkai Zou:** Visualization, Software, Writing – original draft. **Haolin Wang:** Visualization, Software, Writing – original draft. **Hongzhen Tang:** Formal analysis, Visualization. **Zeping Han:** Data curation, Resources. **Wenfeng Luo:** Data curation, Resources. **Jian Shen:** Data curation, Resources. **Fangmei Xie:** Data curation, Resources. **Yi Zhao:** Conceptualization, Writing – review & editing, Supervision. **Zhiming Xiang:** Conceptualization, Writing – review & editing, Supervision. All authors have read and approved the final manuscript.
