# Supplementary material for: CIEC: Cross-tissue Immune Cell Type Enrichment and Expression Map Visualization for Cancer
Source: Genomics Proteomics Bioinformatics. 2024 Oct 3;23(1):qzae067. doi: 10.1093/gpbjnl/qzae067 (PMC12065431; doi:10.1093/gpbjnl/qzae067)
Supplement: qzae067_Supplementary_Data [file qzae067_supplementary_data.zip › supplementary material captions.docx]

# Supplementary material

**Figure S1** **Sample and cell distributions across tissues in each cancer type**

**A.** Sample (left) and cell (right) distributions across tissues in colorectal cancer. **B.** Sample (left) and cell (right) distributions across tissues in lung cancer. **C.** Sample (left) and cell (right) distributions across tissues in esophageal cancer.

**Table S1 The information of all datasets**

**Table S2 Specific marker genes of major immune and non-immune cell types**
