# Supplementary material for: CIEC: Cross-tissue Immune Cell Type Enrichment and Expression Map Visualization for Cancer
Source: Genomics Proteomics Bioinformatics. 2024 Oct 3;23(1):qzae067. doi: 10.1093/gpbjnl/qzae067 (PMC12065431; doi:10.1093/gpbjnl/qzae067)
Supplement: qzae067_Supplementary_Data [file qzae067_supplementary_data.zip › Table S2.docx]

**Table S2 Specific marker genes of major immune and non-immune cell types**

| **Cell type** | **Cell-type specific markers** |
| --- | --- |
| T cell | *CD3D*, *CD3G*, *CD3E*, *CD4*, *CD8A*, *CD8B* |
| B cell | *MS4A1*, *CD79A*, *CD79B* |
| Plasma cell | *MZB1*, *IGHA1*, *IGHG1* |
| Epithelial cell | *EPCAM*, *KRT8*, *KRT18* |
| NK cell | *NCAM1*, *KLRD1* |
| Monocyte/Macrophage/DC | *CD14*, *FCGR3A*, *CD68*, *CD163*, *CLEC9A*, *CD1C*, *CLEC10A*, *LAMP3*, *LILRA4* |
| Fibroblasts | *COL6A1*, *COL1A1*, *THY1*, *DCN* |
| Endothelial cell | *PECAM1*, *CD34*, *VWF* |
| Mast cell | *MS4A2*, *TPSAB1*, *TPSB2*, *KIT* |

*Note*: NK, natural killer; DC, dendritic cell.
