# Supplementary material for: CIEC: Cross-tissue Immune Cell Type Enrichment and Expression Map Visualization for Cancer
Source: Genomics Proteomics Bioinformatics. 2024 Oct 3;23(1):qzae067. doi: 10.1093/gpbjnl/qzae067 (PMC12065431; doi:10.1093/gpbjnl/qzae067)
Supplement: qzae067_Supplementary_Data [file qzae067_supplementary_data.zip › Table S1.docx]

**Table S1 The information of all datasets**

| **GEO accession** | **PubMed ID** | **Reference DOI** |
| --- | --- | --- |
| GSE132465 | 32451460 | 10.1038/s41588-020-0636-z |
| GSE144735 | 32451460 | 10.1038/s41588-020-0636-z |
| GSE146771 | 32302573 | 10.1016/j.cell.2020.03.048 |
| GSE164522 | 35303421 | 10.1016/j.ccell.2022.02.013 |
| GSE166555 | 34409732 | 10.15252/emmm.202114123 |
| GSE178318 | 34489408 | 10.1038/s41421-021-00312-y |
| GSE178341 | 34450029 | 10.1016/j.cell.2021.08.003 |
| GSE188711 | 34793335 | 10.1172/jci.insight.152616 |
| GSE196964 |  |  |
| GSE200997 | 35538548 | 10.1186/s13059-022-02677-z |
| GSE145370 | 33293583 | 10.1038/s41467-020-20019-0 |
| GSE160269 | 38097539 | 10.1038/s41392-023-01710-2 |
| GSE221561 | 37563120 | 10.1038/s41392-023-01518-0 |
| GSE131907 | 32385277 | 10.1038/s41467-020-16164-1 |
| GSE123904 | 32042191 | 10.1038/s41591-019-0750-6 |

*Note*: GEO, gene expression omnibus; DOI, digital object identifier.
