# Supplementary figures and images for: CIEC: Cross-tissue Immune Cell Type Enrichment and Expression Map Visualization for Cancer
Source: Genomics Proteomics Bioinformatics. 2024 Oct 3;23(1):qzae067. doi: 10.1093/gpbjnl/qzae067 (PMC12065431; doi:10.1093/gpbjnl/qzae067)

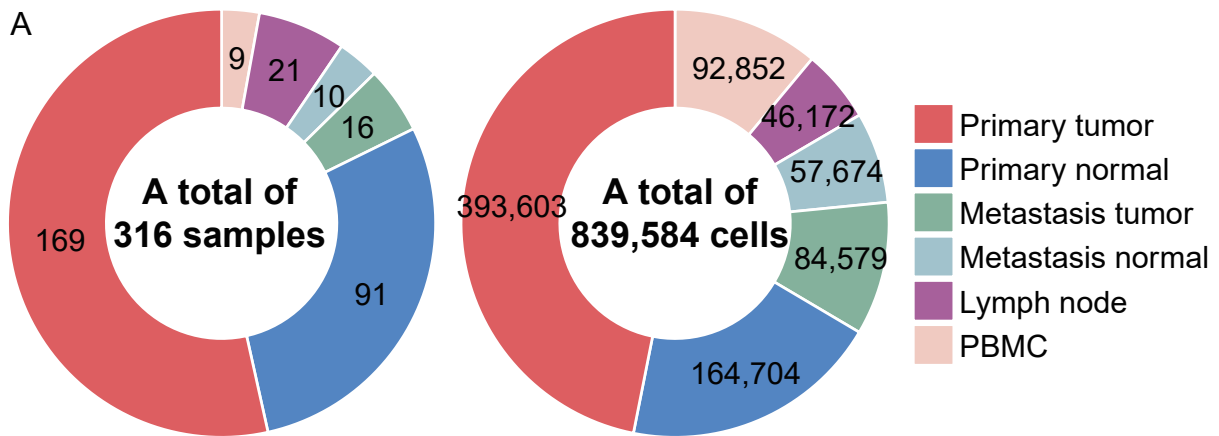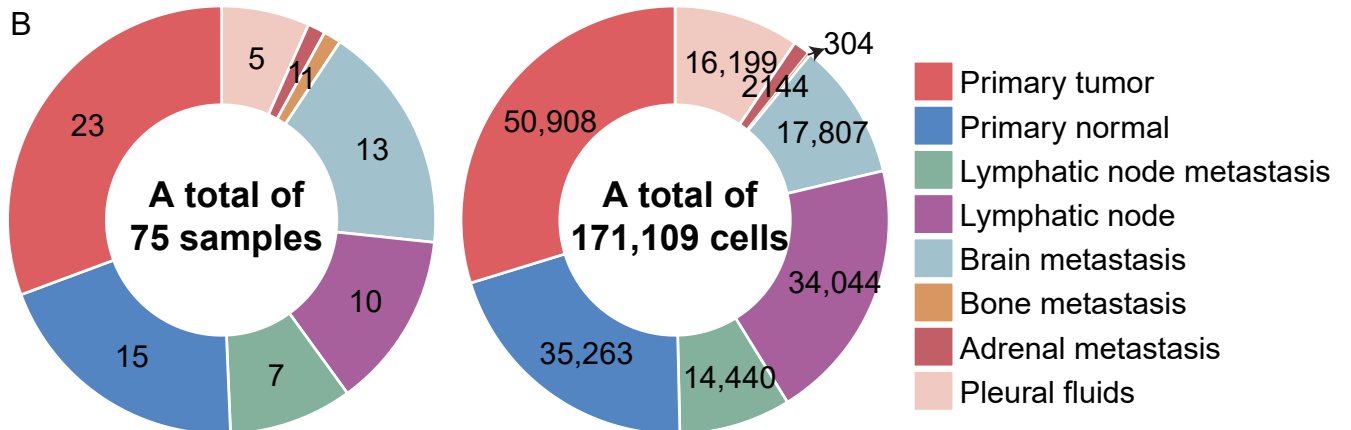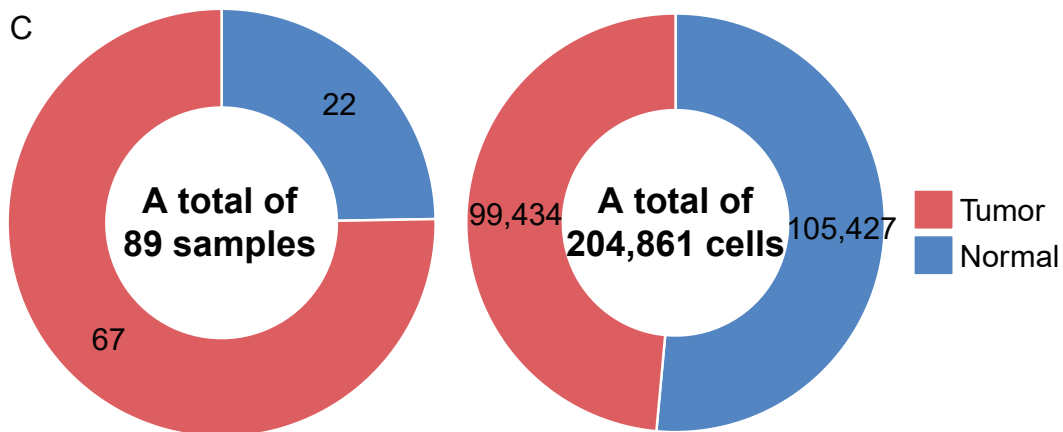

Supplement: qzae067_Supplementary_Data [file qzae067_supplementary_data.zip › Figure S1.pdf]
